# Non-canonical soluble amyloid-beta aggregates and plaque buffering: controversies and future directions for target discovery in Alzheimer’s disease

**DOI:** 10.1186/s13195-017-0293-3

**Published:** 2017-08-17

**Authors:** David L. Brody, Hao Jiang, Norelle Wildburger, Thomas J. Esparza

**Affiliations:** 1 Department of Neurology, Washington University School of Medicine, 660 South Euclid Avenue, Box 8111, St Louis, Missouri 63110 USA; 2 Hope Center for Neurological Disorders, Washington University School of Medicine, 660 South Euclid Avenue, Box 8111, St Louis, Missouri 63110 USA

**Keywords:** Alzheimer’s disease, Dementia, Amyloid-beta, Oligomer, Plaque, Neurotoxicity, Chemical cleavage, Buffering

## Abstract

The specific amyloid-beta (Aβ) species or other amyloid-precursor protein cleavage products that are most directly related to human neurodegeneration and clinical dementia of the Alzheimer’s type have not yet been directly identified. Without a clear understanding of the most relevant species, it is difficult to determine whether therapeutic candidates successfully engaged the correct target(s). Here, we review some of the controversies regarding soluble Aβ aggregates (also termed oligomers, dimers, trimers, Aβ*56, amylospheroids, etc.) and propose experiments designed to move forward towards new therapeutic approaches. Specifically, we review the increasing evidence for the relevance of non-canonical forms of Aβ, the much more potent toxicity attributable to native species than to synthetic Aβ, and the evidence implicating the ratio of soluble Aβ aggregates to plaques in differentiating demented patients from non-demented high Aβ plaque pathology controls. To move forward, we propose four related directions. 1) Narrowing the focus to species derived from human Alzheimer’s disease (AD) brain tissue, as opposed to synthetic Aβ, cell culture-derived species, or species primarily present in animal models. 2) Careful study of differences between patients with dementia of the Alzheimer’s type vs. non-demented controls with high Aβ plaque pathology. This will involve testing the hypothesis that, under some circumstances, plaques may buffer soluble toxic species, but later release them into the surrounding milieu. 3) Investigations of other protein constituents of soluble Aβ aggregates in addition to Aβ itself. Our initial data based on chemical cleavage experiments indicate that other proteins do appear to be part of the human brain soluble Aβ aggregates. 4) Multimodal experimental assessments of toxicity, including longer term effects on synapse loss, related deleterious cellular responses, and degeneration in human-derived neuron-like cells. Overall, the goal is to identify specific Aβ species, other amyloid precursor protein cleavage products, or other key proteins in aggregates present in human AD brains, less abundant in non-demented high pathology control brains, and robustly toxic in a wide variety of relevant assays. These species themselves, the enzymatic or cellular processes involved in their production, and their routes of clearance would be highly relevant therapeutic targets for dementia of the Alzheimer’s type.

## Background

The amyloid cascade hypothesis has been a mainstay of AD research for decades [1]. The genetic evidence regarding familial AD has been interpreted as supporting this hypothesis, as have many pathophysiological mechanistic investigations. However, the specific amyloid-beta (Aβ) species or other amyloid-precursor protein cleavage products that are most directly related to human neurodegeneration and clinical dementia of the Alzheimer’s type have not yet been directly identified. Recent clinical trials in humans ostensibly targeting Aβ have not been successful at alleviating dementia [2], but without clear understanding of the most relevant species and their interactions with their cell biological milieu [3], it is difficult to determine whether these therapeutic candidates successfully engaged the correct target(s). While there are clearly many ways to approach the problem of Alzheimer’s disease (AD), there is some degree of consensus surrounding the following three principles:

1. Genetic linkages in humans using clinical status as the outcome measure represent the highest level of evidence currently available regarding causal relationships in dementia of the Alzheimer’s type. Successful clinical trials would offer a higher level of evidence, but as yet these have not been reported.
2. The Aβ species present in the human brain are the most directly relevant. Synthetic, cell culture-derived, and animal Aβ may not be composed of the most relevant proteoforms and can take a wide variety of aggregation states, not all of which may be present in the human brain.
3. Synapse loss with or without more widespread neurodegeneration is likely to play an important role in clinical dementia. This assertion is based on post-mortem findings in human brain indicating that the extent of synapse loss differentiates patients with dementia of the Alzheimer’s type from high plaque pathology non-demented control subjects [4]. The role of synaptic dysfunction in the absence of synapse loss, neurochemical abnormalities, and other pathophysiological events is not as clear, and has not been as tightly linked to dementia of the Alzheimer’s type.

The most compelling genetic evidence favoring a key role for Aβ or another related amyloid-precursor protein (APP) fragment can be briefly summarized as follows: mutations that cause increases in amyloid precursor protein (APP) production or alterations in cleavage favoring certain products lead to early onset dementia [5]; an APP mutation that attenuates beta secretase cleavage of APP prevents development of dementia [6]; an APP mutation that leads to altered non-fibrillar aggregate forms of Aβ causes early onset dementia [7]; and the genetic polymorphisms that affect proteins involved in lipid metabolism, innate immunity, and other physiological functions modulate risk of dementia, but do not appear to play a directly causal role [8].

This genetic evidence still leaves considerable room for many alternative explanations. While Aβ plaques are one of the key pathological hallmarks of AD, they are not sufficient to cause dementia of the Alzheimer’s type; many cognitively normal elderly individuals have plaques that are as yet indistinguishable qualitatively and quantitatively from those of demented patients [9–12]. Plaques probably do play some toxic role, as there is substantial loss of synapses and disruption of neuronal architecture in their vicinity [13], but this could be due more directly to soluble Aβ species related to the plaques or to microglial processes reacting to the plaques (see below). Furthermore, soluble Aβ monomers are also not likely to play a central role as soluble Aβ monomers are present in the brains of normal individuals at all ages. Despite a great deal of controversy [14], soluble Aβ aggregates have been the leading candidates for toxicity directly relating to dementia in humans [15]. For example, soluble forms of Aβ immunoreactive material that are larger than Aβ monomers appear to be correlated with dementia [16–20]. Human brain-derived Aβ immunoreactive material larger than monomers causes impairments in synaptic plasticity [18] and disruption of neuronal architecture [21]. Apparently different human brain-derived assemblies termed amylospheroids that are reactive to specific antibodies cause neuronal cell apoptosis more potently than similar-sized synthetic Aβ aggregates [22]. However, the precise structures of these toxic species have not been determined. Mass spectrometry has revealed peptide sequences consistent with portions of Aβ, but the results are also consistent with other amyloid precursor protein fragments and not definitive enough to identify the exact species involved.

## What are the characteristics of the putatively toxic soluble Aβ aggregates?

Shankar et al. [18] observed Aβ immunoreactive species that appeared larger than monomers and approximately the hypothetical size of Aβ dimers (7–8 kDa on size exclusion chromatography and SDS-PAGE gels) in aqueous lysates from human AD brains. Preparations containing these putative Aβ dimers caused substantial impairments in long-term potentiation when applied to rodent hippocampal slices and neuritic beading in neuronal cell culture [21]. However, mass spectrometry analyses confirmed only a mid-domain portion of the Aβ peptide. Furthermore, nothing in this size range is apparent using ELISA-type assays sensitive to species with free canonical Aβ N-termini [23, 24]. Several alternative explanations could exist:

1. The species is an Aβ dimer made of two non-canonical Aβ peptides, e.g., extended, truncated, or post-translationally modified forms. The plausibility of this alternative is supported by the recent findings from Portelius et al. [25–27], and our unpublished data indicating substantial non-canonical heterogeneity in Aβ peptide proteoforms in extracts from human brain [28].
2. The species is a non-canonical Aβ peptide stably linked to another molecule that increases its size. Such species have not yet been identified in human brain, but appropriate methods to identify them are lacking.
3. The species is a new amyloid-precursor protein fragment with Aβ mid-domain immunoreactivity but N-terminal and/or C-terminal extensions. Recent evidence for the existence of toxic species in cell culture systems lends plausibility [29–31], but such species in soluble form have not yet been definitively identified in human brain to our knowledge. This is an area that warrants further exploration.
4. The species is a canonical Aβ peptide stably linked to another molecule that increases its size. This initially seems unlikely given that the HJ5.1- HJ3.4 ELISA should detect such species if they are present. However, the other molecule could obstruct the binding of either of the antibodies used in ELISA, even under denaturing conditions.
  Less likely explanations include:
5. The species is a bona fide Aβ dimer made of two canonical Aβ peptides. This seems unlikely given that we and others have not observed a species of this size using sensitive ELISAs that should detect such species. The ELISAs use HJ3.4 and 82E1, antibodies that recognize the free N-terminus of canonical Aβ to both capture and detect Aβ.
6. The species is an Aβ dimer made of one canonical Aβ peptide and one non-canonical Aβ peptide. This seems unlikely given that we and others have not observed a species of this size using a sensitive ELISA that should detect such species. The ELISA uses a mid-domain antibody HJ5.1 to capture and the canonical N-terminus-specific antibody HJ3.4 to detect.
7. The species is an N-terminal canonical but C-terminally extended amyloid-precursor protein fragment. This seems unlikely given that the HJ5.1- HJ3.4 ELISA should detect such species if they were present.

Noguchi et al. [22] reported toxicity of a larger (~150 kDa) species termed amylospheroids derived from human AD brains. These amylospheroids caused apoptosis of mature cultured neurons but not immature neurons or non-neuronal cells. The target appears to be the Na-K ATPase [32]. Matrix-assisted laser desorbtion/ionization (MALDI) mass spectrometry indicated that amylospheriods appear to contain species at the expected molecular weights of Aβ_1-40_ and Aβ_1-42_, but more precise methods such as tandem mass spectrometry that can definitively identify the Aβ species were not performed. Again, many possibilities exist for the identities of the amylospheroid structures.

Lesne et al. reported an approximately 56 kDa species that appears to play a key pathophysiological role in some transgenic mouse models [33] but instead appears inversely related to dementia in humans [34].

In our studies, the soluble Aβ aggregates from human AD brain were predominantly very high molecular weight, eluting close to the void volume of a Superdex 200 size exclusion chromatography column (>670 kDa based on globular protein standards) [23, 24]. Mass spectrometry analyses of partially purified high molecular weight soluble Aβ aggregates revealed full length Aβ sequences [23] as well as many other non-canonical forms of Aβ [28]. As of yet, however, we have not detected specific toxicity of these larger aggregates (unpublished data).

Thus, it appears likely that several discrete types of soluble Aβ aggregates are present in the human brain which may exert different types of toxicity. However, none of the previously reported soluble Aβ aggregate species were assessed explicitly in high plaque pathology non-demented controls, so it is unclear if their concentrations correlate with Aβ plaques or correlate with dementia of the Alzheimer’s type. Furthermore, none of these species have been sufficiently purified to allow accurate structural characterization. Thus, apart from proprietary conformational-specific antibodies, there are no generalizable leads for constructing pharmacodynamic assays or developing targeted therapeutics.

## Relationship between soluble Aβ aggregates and plaques

**Fig. 1:**
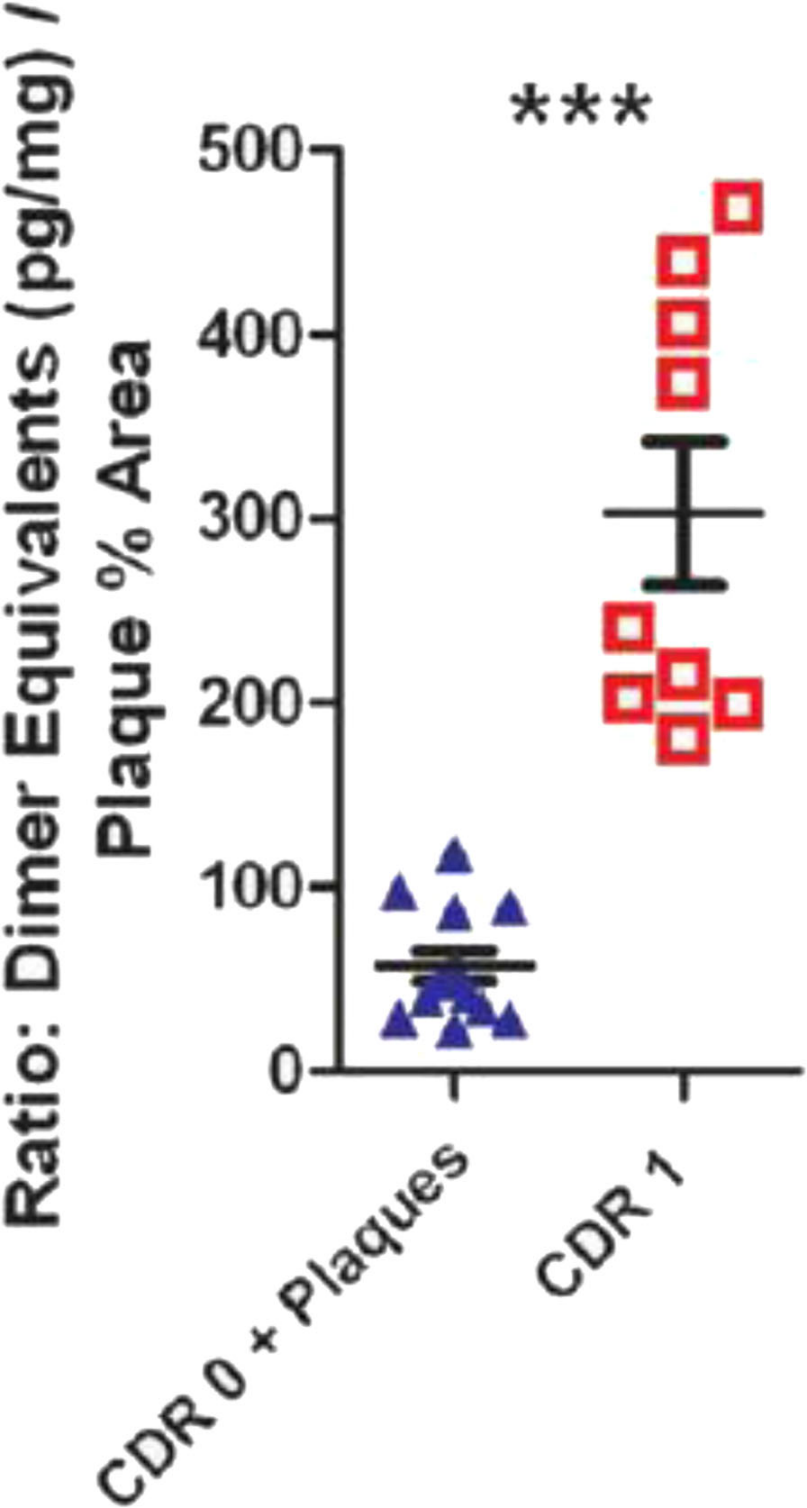
Ratio of soluble Aβ aggregate levels to plaque area fully distinguishes non-demented controls with high plaque pathology from patients with dementia of the Alzheimer’s type and comparable plaque burden. Reproduced from Figure 2N of Esparza et al. 2013 [24]. Soluble Aβ aggregate levels expressed as dimer equivalents (pg/ml). Clinical dementia rating (*CDR*) of 0 indicates absence of dementia and rating of 1 indicates mild/early dementia. ***p = 0.0001, Mann–Whitney U test

**Fig. 2:**
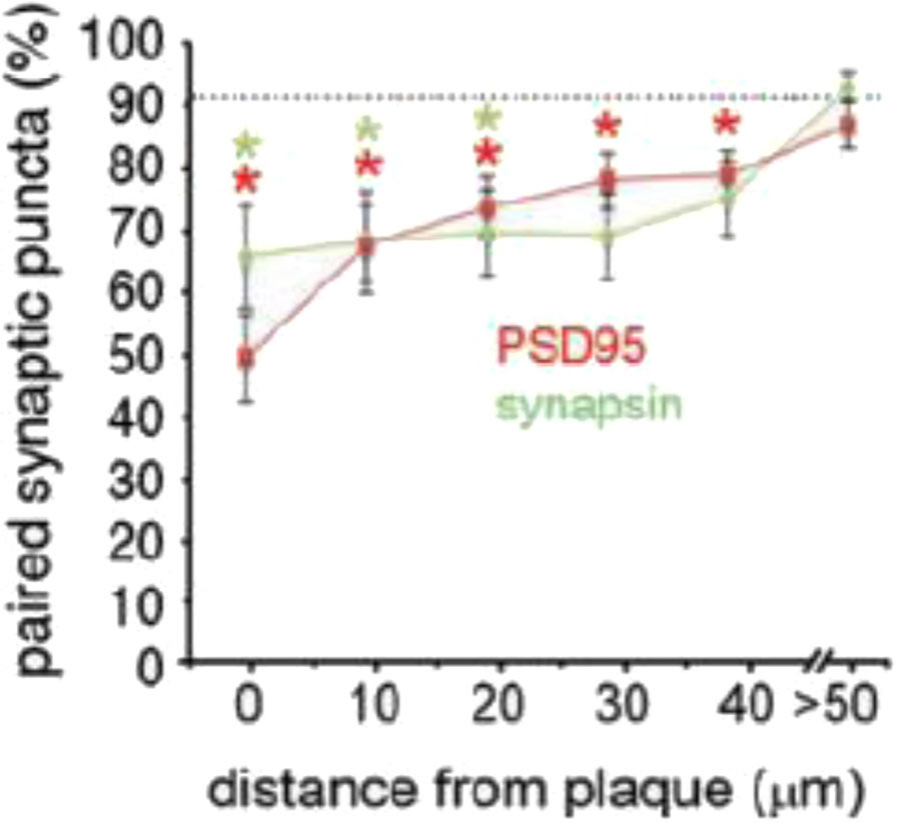
Loss of synapses in human Alzheimer’s disease brain as a function of distance away from plaques. PSD95 is a post-synaptic marker, and synapsin is a presynaptic marker. *Post hoc Wilcoxon *p* < 0.05. Reproduced from Figure 1F of Koffie et al. [13]

We reported in 2013 that the concentration of soluble Aβ aggregates from patients with dementia was higher than the concentration of soluble Aβ aggregates from control subjects with indistinguishable plaque burden but no dementia. The ratio of soluble Aβ aggregate concentration to plaque area fully distinguished these two groups of subjects, with no overlap between groups (Fig. 1, reproduced from Figure 2N of reference [24]). These quantitative measurements were made using an ELISA involving the same N-terminal-specific anti-Aβ antibody to capture and detect, which provided >10,000-fold specificity for soluble aggregates over monomeric Aβ. Spiking soluble monomeric Aβ into the buffer along with brain tissue from plaque-free controls did not result in any detectable soluble Aβ aggregates. The distinction between the ratios of soluble aggregates to plaque burden in tissue from patients with dementia vs. tissue from high plaque non-demented controls was confirmed after replication in a separate cohort. Measurements of overall levels of soluble Aβ (monomeric and soluble aggregates) did not reveal any differences between groups, further indicating a specific relationship with soluble Aβ aggregates.

The interpretation of our findings involving non-demented high plaque pathology controls and patients with dementia, however, is not entirely clear. We and others [35] hypothesized that initially plaques may serve as a reservoir or sink for toxic soluble Aβ aggregates, sequestering them away from other targets in the extracellular space and thereby preventing their toxicity. We posit that over time the reservoir is saturated or loses capacity and the toxic soluble Aβ aggregates become free to diffuse and bind other targets, exerting toxicity and causing dementia. At later stages, the plaques saturated with toxic soluble Aβ aggregates serve as sources releasing soluble Aβ aggregates into the surrounding area and causing a gradient of toxicity to synapses. Support for the idea that plaques can serve as a source of toxicity is provided by the observations of Koffie et al. [13, 36]. They analyzed synapse density as a function of distance from plaques in human AD brains. They found a steep gradient with substantial loss of both pre- and post-synaptic markers near plaques but normal levels >50 microns away (Fig. 2, reproduced from Figure 1F of reference [13]). Interestingly, binding of the antibody NAB61, which recognizes a subset of soluble Aβ aggregates, was also found in a gradient around plaques but the NAB61 gradient decayed over ~20 microns, suggesting that either NAB61-negative species may be playing a role, or that concentrations of the NAB61-positive species were below the limit of detection at greater distances.

## The plaque buffering of soluble Aβ aggregates hypothesis

As stated above, a longstanding hypothesis has been that plaques may buffer soluble Aβ aggregates, protecting neuronal structures from toxicity at early times and then releasing toxic aggregates at later times. The concept of plaque buffering is meant to indicate a generalized functionality. Direct binding and unbinding of soluble aggregates to the plaques themselves would be the most straightforward mechanism, but binding and unbinding to peri-plaque elements such as dystrophic neurites, microglia, and astroctyes would be functionally equivalent. There has been a great deal of interest in specific microglial and astroglial phenotypes induced in the vicinity of plaques [37, 38], and these specific phenotypes could play a role in plaque-related soluble Aβ aggregate buffering capacity. Furthermore, it could be the plaques, the soluble aggregates, or both that change over time.

**Fig. 3:**
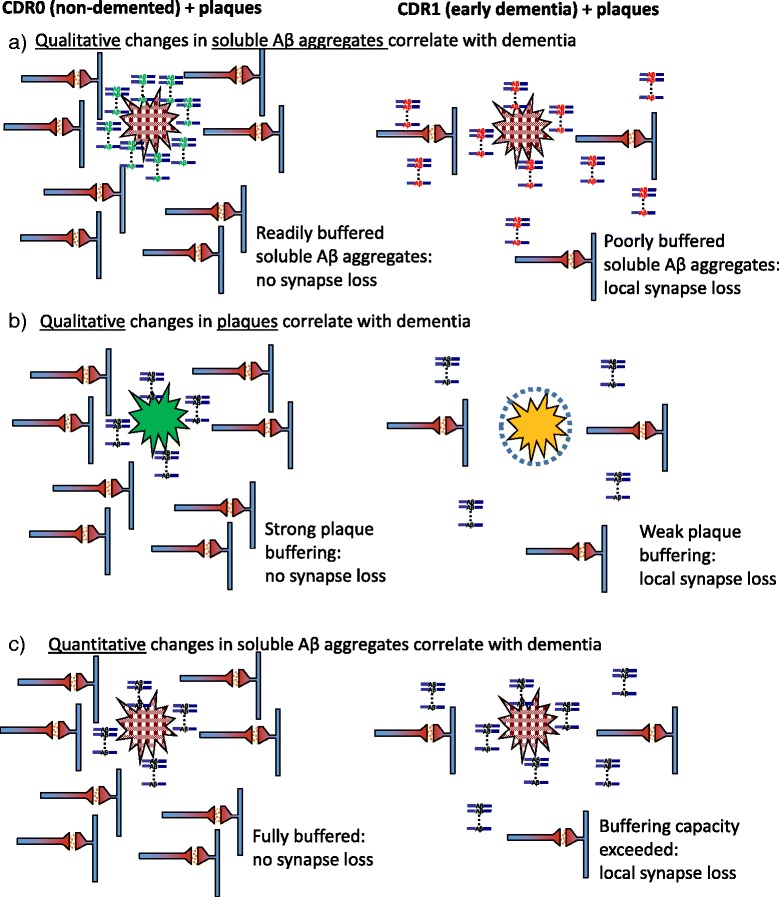
Alternative versions of the plaque buffering of soluble Aβ aggregate hypothesis. The overall hypothesis states that plaques may buffer soluble Aβ aggregates, protecting neuronal structures from toxicity at early times and then releasing toxic aggregates at later times. **a** Sub-hypothesis that *qualitative* changes in the *soluble aggregates* correlate with dementia: The soluble aggregates from non-demented controls could be readily buffered by plaques, but the soluble aggregates from demented patients would not be well buffered by plaques. **b** Sub-hypothesis that *qualitative* changes in the *plaques* correlate with dementia: The plaques from non-demented controls would retain more buffering capacity than the plaques from demented patients. **c** Sub-hypothesis that *quantitative* changes in the *soluble Aβ aggregates* correlate with dementia: The intrinsic buffering properties of soluble Aβ aggregates and plaques from non-demented controls would be similar to those of demented patients when assessed using the same concentrations of soluble Aβ aggregates. (Original figure: D. Brody)

We propose the following line of investigation to test the plaque buffering hypothesis:

1. Develop methods to quantitatively purify relevant soluble Aβ aggregates from human brain.
2. Develop methods to label human brain soluble Aβ aggregates without disrupting their functional properties.
3. In a 2 × 2 design, test the binding of labeled soluble Aβ aggregates isolated from human CDR0 (non-demented) + plaque brains vs. those isolated from CDR1 (demented) + plaque brains on frozen (not fixed) slices from CDR0 + plaque vs. CDR1 + plaque brains. This would allow testing of three specific sub-hypotheses:
  - Key prediction of the sub-hypothesis that *qualitative* changes in the *soluble Aβ aggregates* correlate with dementia: The soluble Aβ aggregates from demented patients would bind to plaques less avidly than soluble Aβ aggregates from non-demented controls (Fig. 3a).

**Table:** 

	Soluble Aβ aggregates	
Plaque containing slices	CDR0 + plaques (non-demented)	CDR1 (demented)
CDR0 + plaques (non-demented)	Strong buffering	Weak buffering
CDR1 (demented)	Strong buffering	Weak buffering
  - Key prediction of the sub-hypothesis that *qualitative* changes in the *plaques* correlate with dementia: The plaques from non-demented controls would retain more buffering capacity than the plaques from demented patients (Fig. 3b).

**Table:** 

	Soluble Aβ aggregates	
Plaque containing slices	CDR0 + plaques (non-demented)	CDR1 (demented)
CDR0 + plaques (non-demented)	Strong buffering	Strong buffering
CDR1 (demented)	Weak buffering	Weak buffering
  - Key prediction of the sub-hypothesis that *quantitative* changes in the *soluble Aβ aggregates* correlate with dementia: The intrinsic buffering properties of soluble Aβ aggregates and plaques from non-demented controls are similar to those of demented patients when assessed using the same concentrations of soluble Aβ aggregates (Fig. 3c).

**Table:** 

	Soluble Aβ aggregates	
Plaque containing slices	CDR0 + plaques (non-demented)	CDR1 (demented)
CDR0 + plaques (non-demented)	Strong buffering	Strong buffering
CDR1 (demented)	Strong buffering	Strong buffering

It is possible that there could be a mixed result: qualitative changes in plaques, *and* qualitative changes in soluble aggregates, *and* quantitative changes in soluble aggregates could correlate with dementia. This would be difficult to fully interpret. A null result is also possible: plaques do not buffer soluble Aβ aggregates. This could indicate a limitation of the methods used or indicate that plaque buffering does not play a major role.

The strong buffering presumes that the endogenous soluble Aβ aggregates associated with the slices have been largely washed out during the preparation of the slices for binding studies. Recent unpublished results from Dominic Walsh’s group indicate that at least some species of endogenous soluble Aβ aggregates do in fact diffuse out of minced tissue (personal communication). The readily diffusible species may represent only a subset of the soluble Aβ aggregates extracted during tissue homogenization.

A technical hurdle will involve labeling the isolated endogenous aggregates without interfering with their binding characteristics. Biotinylation, tritiation, and iodination of N-terminal residues, C-terminal residues, or tyrosine residues could be considered. Competition with unlabeled endogenous aggregates would be an appropriate control to assess the relative binding affinity of labeled vs. unlabeled aggregates. If the labeling protocol alters the characteristics of the endogenous soluble Aβ aggregates, the affinity of binding vs. the affinity of competition would be different. If the affinity of binding and the affinity of competition are found to be similar, this would indicate that the labeling does not markedly alter the endogenous soluble Aβ aggregates. At the same time that plaque buffering capacity is being tested, the endogenous labeled aggregates could also be tested for labeling to other structures such as synapses, microglia, astrocytes, and blood vessels. This could either confirm or refute the relevance of the observation that synthetic Aβ oligomers bind specifically to synapses [39].

The results of these experiments will direct the critical next steps: If qualitative changes in the soluble Aβ aggregates correlate with dementia, an extensive search for the structural differences between soluble Aβ aggregates from controls without dementia vs. soluble Aβ aggregates from patients with dementia would be well justified. Biochemical purification and mass-spectrometric characterization could be appropriate methods. Top-down (undigested) proteomic methods could be used to identify and quantify the specific proteoforms of Aβ in the aggregates from demented vs. non-demented controls [40]. The enzymes responsible for putative truncations and post-translational modifications that correlate best with dementia would be logical targets for new therapeutics. Bottom-up proteomics (following enzymatic digestion) could be used to identify other proteins closely associated with soluble Aβ aggregates from patients with dementia but not associated with soluble Aβ aggregates from non-demented controls. Such co-associated proteins could also be drug targets if they confer or contribute to toxicity. Aβ is amphipathic and could interact with specific lipids, altering its conformation and hypothetically also its toxicity. Mass spectrometry-based lipidomic analyses could be used to identify differentially associated lipids in an analogous manner [41]. The enzymatic processes responsible for the specific lipid synthesis or modification could similarly be drug targets. If, on the other hand, qualitative changes in the plaque buffering capacity correlate with dementia, analysis of the structural determinants in the plaques would be indicated. If qualitative changes are not found in either the plaque buffering capacity or the soluble aggregates themselves, and quantitative differences are implicated, the logical next direction would be to evaluate the mechanisms implicated in the rates of production and clearance of the soluble aggregates.

We envision a scenario in which Aβ production and accumulation are left to proceed (no beta or gamma secretase inhibitors, no immunotherapy), but an enzyme responsible for conversion of a less toxic form of soluble Aβ to a more toxic form is inhibited. The patients, hopefully, would live long lives with brains full of benign plaques but no dementia of the Alzheimer’s type.

## Are other proteins part of the soluble Aβ aggregate complexes?

**Fig. 4:**
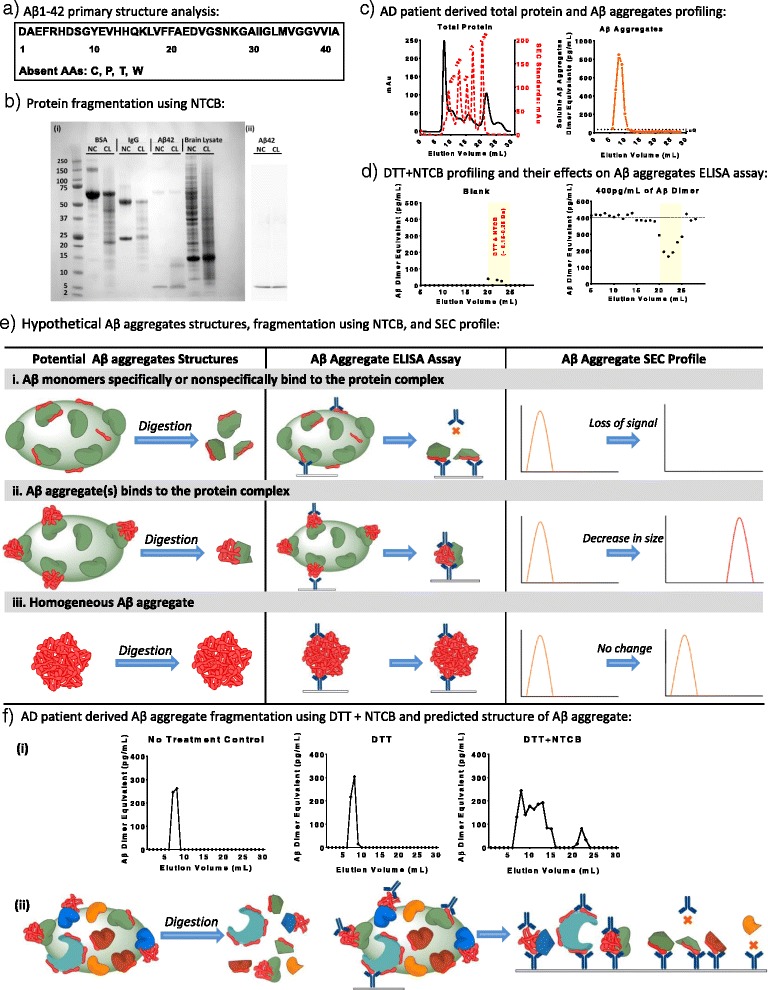
Cysteine cleavage of AD patient-derived Aβ aggregates and predicted structural models of Aβ aggregates. **a** Primary sequence analysis of Aβ_1-42_. Cysteine, proline, threonine, and tryptophan are not present within the 42 residues. **b** Protein fragmentation using 2-nitro-5-thiocyanatobenzoic acid (NTCB). In this study, proteins were pre-treated with 5 mM dithiothreitol (*DTT*) for 30 minutes at room temperature (RT) to reduce cysteine thiols. NTCB at final concentration of 5 mM was then added to cyanylate proteins at cysteine residues for 4 h. After cyanlyation, 2 mM NaOH was used to adjust pH to 9, resulting in cleavage N-terminal to cysteines. The reaction was allowed to proceed for 16 h at RT. The NTCB fragmented proteins were then used for downstream applications directly. (i) SDS-page gel analysis of reduced NTCB fragmented proteins. The positive control bovine serum albumin (*BSA*) and immunoglobulin G (*IgG*) proteins known to contain cysteine residues were fragmented by NTCB successfully, as indicated by the change from non-cleaved (*NC*) to cleaved (*CL*). The negative control synthetic monomeric Aβ_1-42_ was not fragmented by NTCB due to the absence of cysteine. The majority of proteins in AD patient-derived brain lysate were successfully fragmented by NTCB. (ii) Western blot analysis of synthetic Aβ_1-42_ before and after digestion showed no significant difference in size or band intensity. **c** Size exclusion chromatography profiling of total protein and soluble Aβ aggregates from an AD patient using Superdex 200 column. AD patient-derived brain lysate (1 mL) was separated by Superdex 200 column. An ELISA-based soluble Aβ aggregate assay [23] was then used to assess for soluble Aβ aggregates in each fraction. High molecular weight soluble Aβ aggregates were detected in fractions 7 to 10, with the estimated size larger than 670 kDa. **d** Size exclusion chromatography and soluble Aβ aggregate assay profiling of DTT and NTCB alone. DTT and NTCB were added to sample buffer and incubated as described above. DTT and NTCB (1 mL) in sample buffer without any protein was separated using a Superdex 200 column. DTT and NTCB were distributed from fractions 20 to 25, consistent with low molecular weight chemicals (molecular weights of DTT and NTCB are 154.25 and 224.19 g/mol, respectively). Aβ_S26C_ dimer standard was then added to each fraction at a final concentration of 400 pg/mL. There was loss of Aβ dimer standard signal in fractions 20 to 25 but no effect on other fractions. **e** Simplified models of Aβ aggregates and potential outcomes after DTT and NTCB treatment. Model (i): more than one Aβ monomer binds to a protein complex specifically or nonspecifically. In this case, after a successful cleavage N-terminal to cysteine residues with NTCB, the protein complex core will be fragmented into numerous small protein fragments and peptides with no more than one Aβ monomer attached to each fragment/peptide. This would result in loss of signal on the soluble Aβ aggregate assay. Model (ii): one or more low molecular weight Aβ aggregates attach to a protein complex core specifically or nonspecifically, similar to model (i). However, after successful cleavage of cysteine using NTCB, low molecular weight Aβ aggregates attached to smaller protein fragments or peptides can still be detected by the soluble Aβ aggregate assay. Therefore, lower molecular weight Aβ aggregates are expected after a NTCB treatment in model (ii). Model (iii): numerous Aβ monomers aggregate and form a high molecular weight Aβ complex without other proteins. In this case, Aβ aggregates will not be fragmented by NTCB due to the absence of cysteine residues. Therefore, Aβ aggregates will show no change before and after NTCB treatment. **f** Size exclusion chromatography profiling of AD patient-derived soluble Aβ aggregates before and after DTT and NTCB treatment, and predicted structure of AD patient-derived soluble Aβ aggregates. (i) AD patient-derived Aβ aggregate were treated with DTT alone or with DTT followed by NTCB as described above. As shown in (i), DTT alone has no detectable effect on soluble Aβ aggregates; after the treatment with DTT and NTCB, however, the soluble Aβ aggregate assay detected a change in the size distribution of the soluble Aβ aggregates ranging from fraction 7 (>670 kDa) to fraction 16 (approximately 44 kDa). There was a potential false positive signal in fractions 21 to 23 as described above for **d**. The partial cleavage of AD patient-derived soluble Aβ aggregates is most consistent with a more complex structure compared with the three simplified models described in **e**. (ii) Hypothetical structure of soluble Aβ aggregates. After DTT and NTCB treatment, high molecular weight soluble Aβ aggregates appear to have been partially fragmented into various sizes of smaller Aβ aggregates, indicating that proteins with cysteine residues are present in at least some of the soluble Aβ aggregates. The detectable smaller aggregates could contain various types of non-cleavable high molecular weight proteins (or protein fragments) with more than one Aβ monomer attached, or the smaller aggregates could be pure Aβ. Importantly, we cannot determine the extent to which the DTT and NTCB fully cleaved all cysteine-containing proteins. Thus, the continued detection of some high molecular weight soluble Aβ aggregates does not necessarily indicate that these species consist only of Aβ. (Original figure: H. Jiang and D. Brody)

Our initial mass spectrometry data from partially purified Aβ aggregates from human AD brain has indicated that many other proteins are present in these preparations in addition to Aβ (not shown). It could be, of course, that these are impurities that are nonspecifically co-immunoprecipitated. It is also possible that they are true components of the high molecular weight soluble aggregates, which would then be best described as hetero-oligomeric or multicomponent aggregates. This is not an easy question to address using biochemical purification methods alone; the conditions required to obtain very high specificity during the immunoprecipitation may also break up true non-covalent aggregates. An orthogonal approach, upon which we have begun to embark, is to degrade other proteins in a fashion which does not affect Aβ and then ask what effects this has on the size forms of soluble Aβ aggregates from human AD brain (Fig. 4). If the immunoprecipitation pulls down pure Aβ aggregates as well as other proteins that are not part of the same complex, such degradation should not change the size of the Aβ aggregates. On the other hand, if the Aβ aggregates contain other proteins which can be degraded, the Aβ aggregates should shift towards smaller size forms. The amino acid composition of Aβ_1-42_ peptides includes only 16 out of the 20 common amino acids (Fig. 4a). However, we were not able to find proteolytic enzymes that would be expected to have the desired broad cleavage of other proteins but no effects on Aβ. Proline endopeptidase, for example, can carry out non-specific cleavage at other amino acids [42]. Therefore, we instead turned to the chemical degradation literature and found the following chemical cleavage candidates: 2-nitro-5-thiocyanatobenzoic acid (NTCB) has been widely used to cyanlyate and cleave proteins N-terminal of cysteine [43–47]; 5-5′-dithio-bis-(2-nitrobenzoic acid) (DTNB) also cleaves proteins N-terminal of cysteine, though less efficiently than NTCB [44, 46]; N-chlorosuccinimide (NCS) cleaves C-terminal of tryptophan, usually at acidic pH with urea, as the efficiency becomes much lower at neutral and alkaline pH (acidic conditions are known to affect the aggregation state of Aβ, so this was felt to be less optimal [44, 48]); iodosobenzoic acid also cleaves C-terminal of tryptophan at acidic pH [49]; hydroxylamine cleaves between asparagine and glycine, but its efficiency is typically low.

After considerable optimization of conditions, we determined that chemical cleavage using NTCB following cysteine reduction by dithiothreitol (DTT) would be feasible. Control proteins containing at least one cysteine were readily cleaved, whereas synthetic Aβ was not (Fig. 4b). To test whether DTT and NTCB can interfere with the soluble Aβ aggregate assay, the assay was performed on each fraction, either without Aβ dimer standard or with Aβ dimer standard at final concentration of 400 pg/mL. In the absence of any Aβ or other proteins, fractions 21 to 25 with DTT and NTCB showed very weak false positive signals but no effects in fractions 7–20 where Aβ would be expected. When Aβ dimer standard at final concentration of 400 pg/mL was added to each fraction, a significant loss of Aβ dimer standard signal was detected from fractions 20 to 25. The loss of Aβ dimer standard signal was likely due to two factors: 1) the reduction and digestion of the IgGs used for the ELISA by DTT and NTCB, as shown in Fig. 4b; 2) direct digestion of the synthetic mutant Aβ dimer standard, which was produced using Aβ_1-40_S26C containing cysteine in place of serine 26 to produce a disulfide cross-linked dimer. Most importantly, though, these results indicate that DTT and NTCB together have no effect on the soluble Aβ aggregate assay within the range of the high molecular weight Aβ aggregates in fractions 7 to 10. Thus, these chemicals caused some artifacts in the very low molecular mass region, but not in the higher molecular weight regions where soluble Aβ aggregates would be expected to be found after size exclusion chromatography (Fig. 4c, d).

In advance, we specified three possible non-mutually exclusive quaternary structural hypotheses (Fig. 4e). In the first hypothesis, the soluble Aβ aggregates consist of Aβ monomers bound to other proteins but not attached to each other. After digestion of other proteins, the soluble Aβ aggregate ELISA signal would be expected to be lost. In the second hypothesis, the large soluble Aβ aggregates consist of smaller Aβ aggregates in complex with other proteins. After digestion of other proteins, the soluble Aβ aggregates would be expected to decrease in size. In the third hypothesis, the soluble Aβ aggregates consist only of Aβ and any other proteins detected in immunoprecipitation–mass spectrometry may be contaminants. After digestion of other proteins, soluble Aβ aggregates would be expected not to change. Our results were most consistent with a combination of the second and third hypotheses (Fig. 4f). Specifically, after DTT and NTCB cleavage, there was residual soluble Aβ aggregate ELISA signal in the high molecular weight range (fractions 7–9) as well as the appearance of new size forms in fractions 10–15. Thus, the most likely interpretation of these results is that at least some of the native human brain soluble Aβ aggregates include other proteins besides Aβ.

## Moving forward

**Fig. 5:**
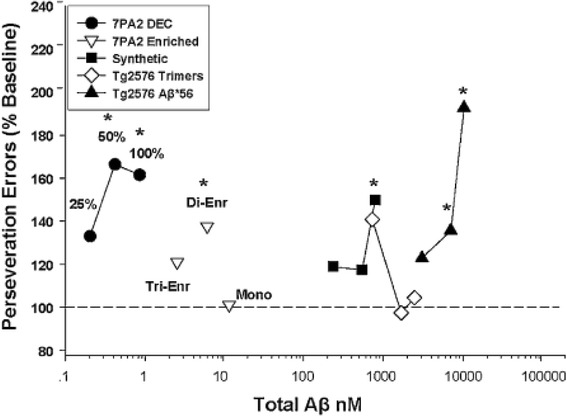
Effects of diverse forms of soluble Aβ aggregates on cognitive performance after intracerebroventricular injection in rats. Reproduced from Figure 4 of Reed et al. [54]. Perseveration errors refer to effects on alternative lever cyclic ratio testing after training to a stable baseline performance. Total Aβ was measured by western blotting with a mixture of Aβ40- and Aβ42-specific antibodies. Unfractionated 7PA2 cell lysates (*7PA2 DEC*) were more potent than fractionated lysates, synthetic oligomers, or species isolated from transgenic Tg2576 mice [33, 61]

Our proposal for moving forward involves assessing the characteristics of the most toxic species present in human brain from patients with dementia of the Alzheimer’s type. The species should correlate with dementia, i.e., they should not be present or occur at lower concentrations in otherwise similar non-demented controls with Aβ plaque pathology. The characterization of the species could start with sensitive immunoassays based on a variety of antibodies to assess the N-terminal extended region, canonical N-terminus, mid-domain, canonical C-terminus, and C-terminal extended regions. We should not assume that they consist of canonical Aβ; many other non-canonical species with substantial aggregation potential and toxicity have been reported [25, 50–52]. ELISAs using the same antibody to capture and detect will be useful for distinguishing dimeric or higher order soluble aggregated species from larger monomeric (e.g., N-terminal or C-terminal extended) species. Mid-domain antibody to both capture and detect Aβ may be more sensitive and more appropriate than ELISAs using the same canonical N-terminus recognizing antibodies; non-canonical forms of Aβ would not be detected by the assays of the type we and others have used previously. Of note, the commercially available oligomer ELISA kit uses the antibody 82E1, which recognizes the free N-terminus of canonical Aβ to both capture and detect and thus suffers from the same “blind spot”. Importantly, detection of a specific species is not sufficient; it should also be determined whether immunodepletion with a specific antibody alleviates toxicity. For example, in Shankar et al. [18], the authors showed that the fractions from AD brain that impaired long-term potentiation had immunoreactivity with antibodies recognizing canonical C-terminal domains. Interestingly, co-administration with 3D6, an antibody recognizing the canonical N-terminus of Aβ, alleviated the impairment of long-term potentiation whereas co-administration with 2G3 and 21 F12, recognizing the canonical C-terminal 40 and 42 termini, did not alleviate the impairment of long-term potentiation. Multiple species with similar size may be present in the same fractions (e.g., several different types of dimers or a mixture of dimers and N- or C-terminal extended species as have been reported in the supernatants from cultured 7PA2 cells or some transgenic mice [29, 31, 53]). Notably, unfractionated 7PA2 cell supernatants are approximately an order of magnitude more potent in inhibiting a sensitive test of cognitive performance in rats than Aβ dimer-enriched or trimer-enriched fractions of 7PA2 cell supernatants [54] (Fig. 5; reproduced from Reed et al. 2011, Figure 4 [54]). Thus, it is possible that an as-yet-uncharacterized species is the primary determinant of behavioral impairment in this system. The relative stability of the species during toxicity assessments should be directly assessed. It is possible that assembly, disassembly, or modification of the species could be occurring during the toxicity assays and could alter the toxicity. Critical controls required will include ruling out the possibility that the toxic species form during the extraction and purification process due to assembly or disassembly [23, 55].

There may be more than one major class of toxic soluble Aβ aggregates. Liu et al. [56] proposed that there are at least two, type 1, which are not closely associated with plaques, and type 2, which are abundant and plaque-associated but not toxic. These categorizations were based on findings in transgenic mice, however, and the extent to which analogous relationships hold in human brain has not been well-defined. Taking the results from multiple labs cited above together, it appears likely that there are more than two types of soluble Aβ aggregate in human brain. Interestingly, however, various size forms may interconvert under some circumstances. Yang et al. [55] reported recently that incubation of high molecular mass relatively non-toxic soluble Aβ aggregate in alkaline ammonium acetate converts them into smaller, more toxic forms. We reported a similar phenomenon as a potential explanation for why size exclusion chromatography in ammonium acetate appeared to give different results to size exclusion chromatography in pH 7.4 saline solutions [23]. Whether this interconversion occurs physiologically in the human brain has yet to be determined.

A critical question in the field is which in vitro or in vivo model system best recapitulates the key aspects of neurodegeneration underlying dementia of the Alzheimer’s type. The mainstay of investigations into neurodegenerative effects have involved assessments of cell and synapse loss in rodent neuronal cell culture systems, effects on synaptic plasticity and structure in rodent brain slices, and behavioral and morphological assessments in animal models. None of the typical toxicity assays involve human cells. De Strooper’s group recently demonstrated that human induced pluripotent stem cell (IPSC)-derived neurons were more susceptible to degeneration than mouse IPSC-derived neurons when transplanted into the brains of APP transgenic immunodeficient mice [57]. This system, and the organoid human IPSC-derived system from the Tanzi lab [58], may represent important, though relatively modest, throughput, avenues for toxicity assessments of human brain-derived soluble Aβ aggregates. The question of temporal scale is also salient. How can neurodegenerative processes proceeding over decades be accurately modeled on an experimentally tractable timeline? Proof-of-concept longer timeline experiments compared mechanistically with shorter timeline experiments using higher concentrations of candidate brain-derived toxic species could be informative. Injection of candidate human brain-derived species into non-human primate brain for assessment of synapse loss, as has been done with synthetic species [59], could also be very useful as proof-of-concept. Non-human primate studies are clearly not feasible for screening and other assessments requiring moderately high throughput.

Once the characteristics of the most toxic species present in human AD brain have been determined, quantitative methods for tracking the species during purification should be employed; an ELISA or similar relatively high throughput assay would be most appropriate. Quantitative bookkeeping should be used to ensure that the purified species represent the predominant species, rather than a small minority species that is selectively enriched. Then, after purification, high resolution tandem mass spectrometry could be used to specifically identify non-canonical forms of Aβ [60], N-terminal extended peptides, C-terminal extended peptides, and other co-associated molecules.

## Conclusions

Looking forward into the future, if one or more human brain toxic species can be specifically characterized, a critical next step will be to develop pharmacodynamic assays for use in human patients. With good pharmacodynamic assays, quantitative assessment of target engagement in early phase clinical trials can be performed to guide dosing in human patients. Without good pharmacodynamic assays, it will be impossible to tell whether candidate therapeutics actually cross the blood–brain barrier and engage the target. In our view, none of the failed clinical trials in AD have compellingly demonstrated target engagement, irrespective of the question of whether they were targeting the right target. Thus, while some have stated that the amyloid hypothesis is on its last legs, it may be more correct to state that it has not yet been sufficiently tested.

## Abbreviations

AD: Alzheimer’s disease
APP: Amyloid precursor protein
Aβ: Amyloid-beta
BSA: Bovine serum albumin
CDR: Clinical dementia rating
DNTB: 5-5′-Dithio-bis-(2-nitrobenzoic acid)
DTT: Dithiothreitol
ELISA: Enzyme linked immunosorbent assay
IgG: Immunoglobulin G
MALDI: Matrix-assisted laser desorbtion/ionization
NCS: N-chlorosuccinimide
NTCB: 2-Nitro-5-thiocyanatobenzoic acid
